# How Working Memory Provides Representational Change During Insight Problem Solving

**DOI:** 10.3389/fpsyg.2018.01864

**Published:** 2018-10-01

**Authors:** Sergei Korovkin, Ilya Vladimirov, Alexandra Chistopolskaya, Anna Savinova

**Affiliations:** ^1^Department of Psychology, Yaroslavl State University, Yaroslavl, Russia; ^2^Laboratory for Cognitive Studies, The Russian Presidential Academy of National Economy and Public Administration, Moscow, Russia

**Keywords:** insight, working memory, representational change, probe-task, executive functions, storage and control systems

## Abstract

Numerous studies of insight problem solving are focused on both the control and storage systems of working memory. We obtained contradictory data about how working memory systems are involved in insight problem solving process. We argue that measuring the dynamics of the control system and storage systems through the course of problem solving can provide a more refined view on the processes involved, as a whole, and explain the existing controversies. We theorize that specific insight mechanisms require varying working memory capacities at different stages of the problem solving process. Our study employed a dual task paradigm to track the dynamics of working memory systems load during problem solving by measuring the reaction time in a secondary probe-task during different stages of problem solving. We varied the modality (verbal, visual) and the complexity of the probe-task during insight and non-insight problem solving. The results indicated that the dynamics of working memory load in insight problems differs from those in non-insight problems. Our first experiment shows that the complexity of the probe-task affects overall probe-task reaction times in both insight and non-insight problem solving. Our second experiment demonstrates that the solution of a non-insight problem is primarily associated with the working memory control system, while insight problems rely on relevant storage systems. Our results confirm that insight process requires access to various systems of working memory throughout the solution. We found that working memory load in non-insight problems increases from stage to stage due to allocation of the attentional control resources to interim calculations. The nature of the dynamics of working memory load in insight problems remains debatable. We claim that insight problem solving demands working memory storage during the entire problem solving process and that control system plays an important role just prior to the solution.

## Introduction

For a long time, the problem of working memory role in problem solving, particularly in insight problems, was (and still is) a focus of numerous studies in the field. A number of reviews and original research articles have been devoted to working memory in problem solving (Hambrick and Engle, 2003; Wiley and Jarosz, 2012). An interest in the role of working memory during insight problem solving stems from the information processing theories viewing insight as a representational change that can possibly occur within working memory (Ohlsson, 1992, 2011; Öllinger et al., 2013). Baddeley’s working memory model describes both the storage systems (visuo-spatial sketchpad, phonological loop and episodic buffer) required to hold representations and the control system (central executive) enabling the restructuring process (Baddeley, 2002). Investigating the processes involved in working memory during problem solving can provide a unique perspective into its internal structure. The conclusions drawn from the working memory studies can be useful for answering the vital question: “Are there any specific mechanisms dedicated to insight solutions?”

Information processing theories seek to determine whether there is something special in insight phenomenon that makes it uniquely different from analytical problem solving; whether insight is a metacognitive epiphenomenon accompanying a broad range of unrelated processes, or whether it involves specific cognitive mechanisms. At first sight, comparing the information processing occurring in different types of problems is a good way to approach this question. Although this widespread approach seems encouraging, studies that employ the traditional experimental designs and paradigms commonly used in working memory research (e.g., distractors in the dual task paradigm, working memory span studies) often report controversial results.

### Contradictions in Working Memory Effects

A number of studies have revealed contradictory results regarding the role of working memory in insight problem solving process (DeCaro et al., 2016, 2017; Chuderski and Jastrzȩbski, 2017). The discussion on the role of working memory in insight primarily focuses on the working memory control system in problem solving. Some studies claim that working memory is a crucial component of both insight and non-insight problem solving processes. Working memory capacity has a strong positive correlation with insight problem solving performance and creativity (Cinan and Doğan, 2013; Chuderski, 2014; Chuderski and Jastrzȩbski, 2018). De Dreu et al. (2012) demonstrated that creative task performance suffers under working memory load. DeYoung et al. (2008) showed that insight problems are as related to working memory as non-insight problems, but only insight problem solving is related to divergent thinking and breaking the frame. Murray and Byrne (2005) found that accuracy in insight problem solving is positively correlated with working memory storage as well as with attention switching processes, but not with selective and sustained attention. However, some studies revealed different effects of working memory control and storage systems on insight problems. Nęcka et al. (2016) claimed that insight problem solving positively correlates with the recognition of the already presented items in working memory (updating processing in working memory storage) rather than with the substitution of old items with new ones (executive control).

Other studies revealed that working memory affects insight problems less than non-insight problems. Concurrent counting during the problem solving process shows a greater negative effect on non-insight than insight problems, and these findings were supported by ERP data via P300 amplitude analysis (Lavric et al., 2000). Ash and Wiley (2006) demonstrated that insight problems with reduced initial phase are not as related to working memory. Fleck (2008) found that insight problem solving correlates only with verbal working memory, but not with control system or spatial working memory. Verbal working memory may affect only the initial phases of problem comprehension without affecting specific insight processes.

Some studies clearly demonstrated that working memory deficits can be beneficial to insight problem solvers. For example, lateral frontal lobe damage patients solve matchstick problems better compared to healthy participants (Reverberi et al., 2005). Participants with mild alcohol intoxication perform remote associate tests better, faster, and experience more insight solutions (Jarosz et al., 2012). Higher working memory capacity is associated with lower matchstick problem accuracy due to inhibited constraint relaxation (DeCaro et al., 2016). Additionally, higher working memory also leads participants to employ complex ineffective strategies in water jar tasks despite the availability of simpler strategies (Beilock and DeCaro, 2007).

Moreover, there is different data regarding the role of storage systems of working memory in insight problem solving. Performance in insight problem solving is not linked to the control system but is associated with the verbal and visuo-spatial components of working memory (Gilhooly and Fioratou, 2009). Gilhooly and Murphy (2005) claimed that verbal insight problem solving rates are positively related to verbal working memory (vocabulary scores) and spatial insight problem solving rates are positively related to spatial working memory (spatial flexibility). Performance on the nine-dot problem is related to spatial but not verbal working memory (Chein et al., 2010). However, the storage systems of working memory are not involved in insight problem processing independently of the control system. Performance in Compound Remote Associate problems can be predicted by both verbal working memory and attention switching (Chein and Weisberg, 2014). On the other hand, verbal working memory distraction via articulatory suppression enhances insight problem solving because it reduces the verbal-based problem processing (Ball et al., 2015). Surprisingly, the preliminary load of spatial working memory enhances the solution rate in the T-puzzle insight problem (Suzuki et al., 2014).

Some controversies can be accounted for by the differences in the procedures and task materials used in these studies. However, the main source of these controversies might stem from two other major factors: heterogeneity of the problem solving process and the complex nature of the working memory model.

Heterogeneity refers to the idea that insight problem solving process consists of several phases (problem comprehension, impasse, and representation restructuring) that are not equally related to working memory. For example, the selective forgetting hypothesis claims that forgetting and memory clearing occurs during the impasse phase (Simon, 1977; Ohlsson, 1992). According to this hypothesis, reduced attention control should be less demanding on the control system of working memory during the impasse phase compared to other phases. The relationship between working memory and insight problem solving can change from phase to phase during this process (DeCaro et al., 2017). The dynamics of insight problem solving processes are infrequently discussed within the working memory studies (Ash and Wiley, 2006; Korovkin et al., 2014; Yeh et al., 2014; Lv, 2015). At the same time, heterogeneity of the phases in insight problem solving was demonstrated in eye-movement studies (Knoblich et al., 2001; Ellis et al., 2011; Yeh et al., 2014). Thus, we propose that the role of working memory in problem solving should be discussed in regards to each phase separately.

The working memory model itself is a challenging theoretical framework featuring certain ambiguity in terms of relevant components and parameters. This challenge is aggravated by the lack of unity between theoretical models of working memory (Engle et al., 1999; Baddeley, 2002; Cowan, 2010). Two main approaches to working memory studies in problem solving are experimental and individual differences approaches (Hambrick and Engle, 2003). These approaches differ not only in their methodology but also in their theoretical basis. The experimental approach typically incorporates the distraction paradigm and is based on Baddeley’s (2002) working memory model. Distractors selectively target one of the storage systems of working memory to isolate the modal-specific effects within the problem solving process. The individual differences approach is based on the concept of working memory capacity and focuses on the quantity of stored items. We consider it necessary to take all characteristics of working memory into account to shed light on the processes that make up insight. Understanding the control system is crucial to describing overcoming of the impasse. Additionally, understanding the modal-specific storage systems is necessary to reveal the mechanisms of representation restructuring. Finally, understanding the overall capacity is essential for assessing the information processing aspects of problem solving.

### Probe-Task

Conventional methods used in working memory studies do not capture the dynamics of working memory load over time. We propose a technique that can accomplish this goal. This technique relies on the assumptions drawn from Kahneman’s (1973) resource model. According to this model, cognitive resources are limited and distributed in concordance with subjective importance. Therefore, if two tasks are performed at the same time continuously, the performance drop in one of them, indicating that available resources have been allocated to the second task instead. If participants should engage in problem solving, while performing a monotonous secondary probe-task, the reaction time in the probe-task should increase whenever the primary problem solving process becomes particularly resource demanding, and vice versa.

Wieth and Burns (2014) clearly showed that both insight and non-insight problem solving processes suffer under multitasking conditions. This fact is in line with our assumptions that the problem solving process competes with the secondary task for resources. Moreover, the interference which occurs due to the competition does not appear to be very damaging to the problem solving process. The surprising result is that providing an incentive does not allow participants to overcome the difficulties associated with multitasking. This may be due to limited attentional resource which cannot be significantly increased. Instead, the authors assume that high motivation leads to surface processing. This means that in the multitasking condition participants shift their attention to the simpler task, essentially making the secondary task the main task. This fact could be a limitation when only using reaction times as the only dependent variable in a dual-task paradigm. Thus, we used reaction times as a main dependent variable and solution rates, solution times, and probe-task accuracy as additional indicators.

The overall problem-solving trial time can be divided into several equal time stages. For example, if the problem was solved in 300 s, the data obtained within the first 100 s, middle 100 s, and last 100 s would represent three stages and corresponding dynamics. Splitting this process into three stages allows us to trace the temporal dynamics of working memory.

Based on the assumption that working memory resources are not unified, we can also vary the content of the secondary probe-task in such a way that it should compete with only some of the systems, but not others. For example, by varying the overall complexity of the probe-task we can investigate the overall working memory capacity demands in problem solving, while, by altering the content of the probe-task (e.g., modality of stimuli) we can isolate the effect of specific storage systems availability.

This technique allows us to answer the following questions on the role of working memory during the insight problem solving process:

(1) Is working memory necessary for insight problem solving process? Does working memory load vary across insight and non-insight problems? Does the insight problem solving process add to working memory load in addition to single probe-task performance?
(2) Are working memory storage systems, the control system, and their overall capacities that are involved in insight problems drastically different compared to non-insight problem solving?
(3) Is there a specific pattern of the temporal dynamics of working memory load during the insight problem solving process? Do capacity, storage, and control systems demands differ across various phases of problem solving?

The study described below was designed to answer these questions regarding the role of working memory and its components in insight problem solving. It was operated under the aforementioned assumptions associated with the dual-task paradigm. This allowed us to operationalize the level of working memory load (low/high) caused by the problem solving process via the reaction time in the simultaneously performed probe task; the slower the reaction time, the higher the working memory load.

## Experiment 1

Experiment 1 was conducted to test hypotheses about the role of working memory in insight problem solving. First, we hypothesized that working memory is necessary for insight problem solving; although not to the same degree as for non-insight problem solving. We predicted that working memory load in insight problem solving will be significantly greater than baseline yet significantly lower than in non-insight problem solving. Second, we expected the probe-tasks to take up the working memory capacity proportionally to their complexity. Third, we predicted that different stages of the problem solving would require different amounts of working memory; more specifically, working memory load should be higher toward the end of problem solving in both problem types due to the accumulation of problem-related information.

To test these hypotheses, we employed a 2 (problem type) × 2 (probe type) × 3 (problem stage) full factorial within-subject design with the reaction time in the probe task serving as a dependent variable. The problem type variable consisted of two levels: insight problems and non-insight problems. The probe type variable featured two levels varying in the number of items held in working memory: a simple probe-task (two possible choices) and a complex probe-task (six possible choices). The problem stage acted as a grouping variable with three levels: the average reaction time in the probe task during the first, the middle, and the last part of overall problem solving time course. Full factorial design was incorporated leading to four (2 × 2) conditions that were later split into three stages each.

### Method

#### Participants

Participants in the experimental group were 32 people (25 women), aged 18–34 (*M* = 22.16; *SD* = 3.18). Participants in the control group were 32 people (22 women), aged 18–28 (*M* = 21.66; *SD* = 2.61). The majority of the sample consisted of undergraduate and graduate students at Yaroslavl State University. All participants were tested individually, took part voluntarily, and were not paid for their participation.

#### Stimuli

We had two types of probe-tasks:

##### The Simple Probe-Task

Participants were shown the pictures of two alternatives: a circle and a square. Participants were instructed to respond by pressing the left key if they saw a circle and the right key button if they saw a square. The participants’ goal was to perform the task as quickly and accurately as possible.

##### The Complex Probe-Task

Participants performed the same task, but had six alternatives choices instead. The alternatives were: a square, a circle, a triangle, a cross, a pentagon, and a hexagon. Participants were instructed to press the left key if they saw a circle, a triangle or a pentagon, and the right key in all the other cases.

All probe-tasks were presented in the center of the screen. All figures were black; the background was white. All trials were preceded by a brief (100 ms) blank screen. These probe-tasks were designed to be demanding, yet realistically possible to be performed simultaneously with the primary problem.

We used two types of problems as a primary task:

##### Non-insight Problems

These problems have clear conditions, a solution algorithm and a logical answer. Participants know all important operators for finding a correct solution and have the right representation of conditions. An example of a non-insight problem: “Given four coins of identical look and feel, two of which are slightly heavier and two are slightly lighter, how could one identify all of them when only allowed to use the balance scale twice?”

##### Insight Problems

These problems require a change of operators or representation, wherein the participant does not know a new system of operators. The solution occurs suddenly and is often associated with an emotional response. An example of an insight problem: “If you have black socks and brown socks in your drawer, mixed in a ratio of 4–5, how many socks will you have to take out to make sure that you have a pair the same color?”

We selected problems with average solution time between 60 and 150 s. In this experiment we used verbal problems only. Participants were not allowed to use notes and write any information down because this would conflict with the probe-task performance. The problems were solved aloud, and participants answered verbally. All the problems are presented in the **Supplementary Materials**. The control group (no probe-task) was included in this study to verify whether or not problem solving was substantially altered by the dual-task itself and whether probe-task performance is affected by the problem solving process in the first place. Participants in the control group solved the same set of problems as in the experimental group but without any secondary task (4 insight and 4 non-insight problems).

The experiment was performed with PsychoPy2 scripts (Version 1.81.02; Peirce, 2008) on the HP Envy x360 15-ar001ur computer with a 15.6″ screen.

#### Procedure

**FIGURE 1:**
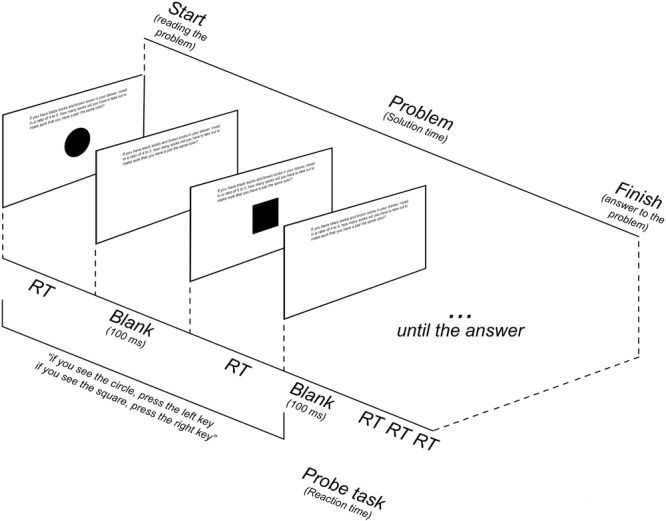
The scheme of the experimental procedure.

Each participant completed two parts of the experiment: practice trials and experimental trials. The purpose of the practice trials was to familiarize participants with the secondary probe-tasks. During the practice trials participants completed 30 trials of both types of probe-tasks – one at a time, not engaged in the problem solving process. There were 30 trials of each type of probe-tasks presented in random order. Average reaction time of the probe-tasks was calculated and served as a baseline for future comparisons. The scheme of the procedure is presented in **Figure 1**.

When participants finished the practice trials, they proceeded to the experimental trials. Each participant solved two insight and two non-insight problems per each of two probe-task levels in random order (eight problems total). The probe-task trials repeated indefinitely for as long as it took to finish the primary problem. Participants had up to 5 min to solve each problem and were instructed to report the proposed solution verbally. Unsolved trials were not included in the data analysis. Participants were provided with a short break (up to 1 min) after each problem trial.

#### Preliminary Analysis

Each of the 32 participants in the experimental group attempted to solve 8 problems (256 problems in total). Trials in which participants solved the problem in under 30 s were excluded from the analysis, since such a short thinking time might be indicative of participants’ exposure to a given problem in the past. Trials that took more than 5 min were considered unsolved and were excluded as well. Besides those exclusions, extreme values of the probe-task reaction times above 3 IQR were considered indicative of participant’s low engagement in the task and, therefore, were excluded from the analysis. Overall, 15 non-insight trials and 50 insight trials were excluded from the analysis. The rest of the trials constituted the obtained data set. The control group data was pre-processed the same way: 9 non-insight trials and 51 insight trials were excluded.

Each problem solving trial was split into three equal time intervals similar to the approach previously used by Knoblich et al. (2001). After that, we averaged the probe-task reaction time within each of those stages, resulting in three probe-task reaction time observations per problem trial. Data obtained from problems in the same condition were averaged across participants, giving us a single data point per each condition for each participant.

The decision to split the overall solution time into three stages was the result of a compromise: while having only two stages would insufficiently represent the course of the problem solving process since it would leave the middle stage of the problem solving unobserved; having more stages can lead to over-conservative statistical estimations due to the aggressive multiple comparison correction, making it hardly possible to reach significance even with a profound effect. We consider the division into three stages theoretically plausible as well: the first stage represents the familiarization with a problem, the middle stage is representative of an impasse, and the final stage is related to overcoming the impasse as well as solution verification.

### Results

The preliminary analysis revealed that participants typically successfully solve the majority of the problems (the average solution rate is 77.9%). Participants were successfully performing the probe-tasks as well (95.7% accuracy). This data suggests that participants were adequately focused on both the primary problem and secondary probe-tasks. We found that there are no significant differences between the control and experimental groups in solution times, *F*(1,62) = 0.004, *P* = 0.952, $\eta_{p}^{2}$< 0.001; there is no main effect of problem type, *F*(1,62) = 0.565, *P* = 0.455, $\eta_{p}^{2}$< 0.009; as well as no interaction between the group and problem type factors, *F*(1,62) = 0.163, *P* = 0.687, $\eta_{p}^{2}$= 0.003. We, therefore, argue that the probe-task does not substantially alter the problem solving process itself. Despite the difference between the solution rates of insight and non-insight problems, we suggest that the difficulty of problems has no major effect on reaction time because for both problem types, only trials of the approximately same duration (30–300 s) were analyzed. A brief overview of these results can be found in **Table 1**. For a detailed analysis refer to the **Supplementary Table S4**.

**Table 1:** The descriptive statistics of solution time and solution rate of the problems in Experiment 1.

	Control group				Experimental group			
	<30 s	>300 s	Solution rate	Solution time, sec (*SD*)	<30 s	>300 s	Solution rate	Solution time, sec (*SD*)
Insight problems	31 (24.22%)	20 (15.625%)	77 (60.16%)	105.47 (48.88)	22 (17.19%)	24 (18.75%)	82 (64.06%)	107.98 (46.01)
Non-insight problems	3 (2.34%)	6 (4.69%)	119 (92.97%)	102.04 (34.82)	3 (2.34%)	4 (3.125%)	121 (94.53%)	110.52 (34.66)

*<30 sec, number of previously known problems or problems solved in less than 30 s and excluded from the further analysis. >300 s, number of problems solved in more than 5 min and excluded from the further analysis.*

**FIGURE 2:**
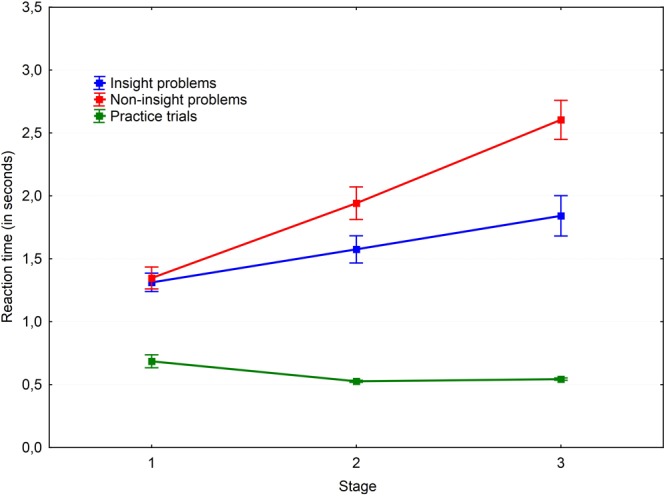
Dynamics of working memory load via the simple probe-task. Vertical bars denote standard errors.

**FIGURE 3:**
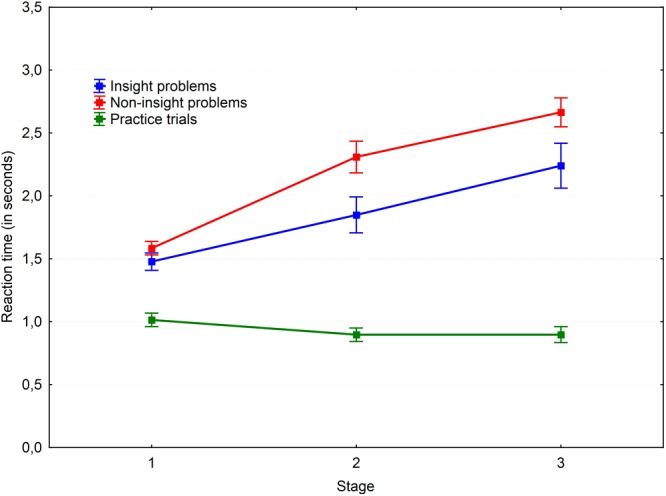
Dynamics of working memory load via the complex probe-task. Vertical bars denote standard errors.

A 3 × 2 × 3 repeated measures ANOVA with Greenhouse–Geisser correction was performed to test our hypotheses. The results are shown in **Figures 2, 3**. A main effect of the probe-task type was found for reaction time, *F*(1.94,40.72) = 184.18, *P* < 0.001, $\eta_{p}^{2}$= 0.898. *Post hoc* pairwise comparisons with the Bonferroni adjustment revealed that reaction time in all three groups were significantly different. The fastest condition was the practice trials with a single probe-task without parallel problem solving (*M* = 0.79; *SD* = 0.15); the slowest condition was non-insight problem solving with a parallel probe-task (*M* = 1.93; *SD* = 0.43). The difference between the practice trial and non-insight problem conditions was found to be significant [*t*(27) = -14.83, *p* < 0.001, *r* = -0.874]. The probe reaction time in the insight problem condition (*M* = 1.67; *SD* = 0.42) was significantly greater than in practice trials [*t*(28) = 12.97, *p* < 0.001, *r* = 0.828] and significantly less than in non-insight problems [*t*(28) = -4.32, *p* < 0.001, *r* = -0.319]. Thus we may conclude that insight problem processing competes with the probe-task for resources of working memory. This means that working memory is necessary for insight problem solving, but is not as crucial for non-insight problem solving.

**FIGURE 4:**
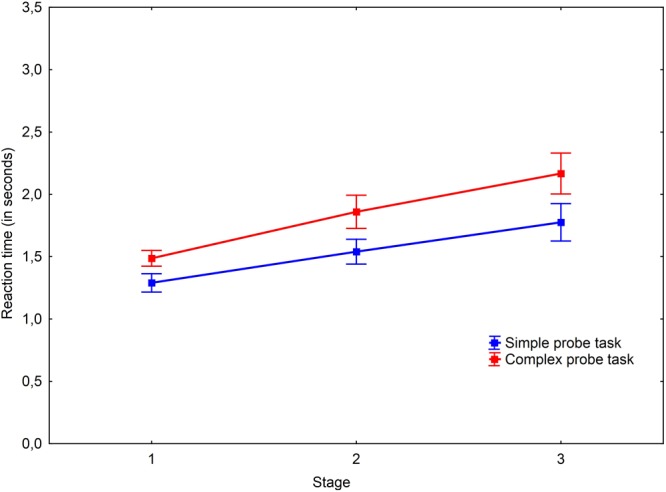
Dynamics of working memory load in the insight problems. Vertical bars denote standard errors.

**FIGURE 5:**
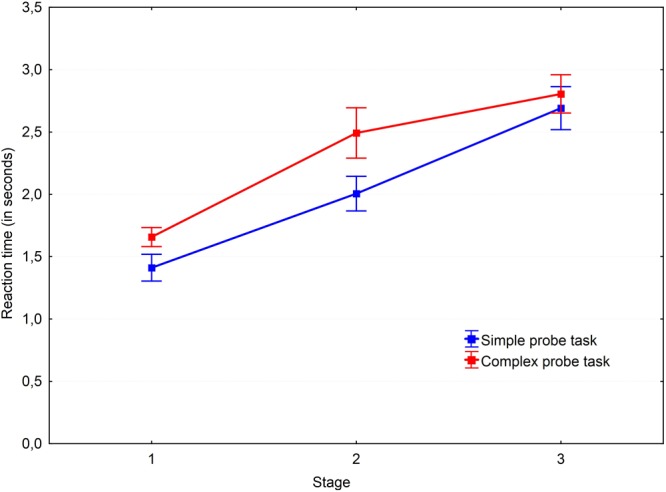
Dynamics of working memory load in the non-insight problems. Vertical bars denote standard errors.

A main effect of probe type was revealed [*F*(1,21) = 32.65, *P* < 0.001, $\eta_{p}^{2}$= 0.609]. The results are shown in **Figures 4, 5**. *Post hoc* analysis of the probe-tasks in practice trials showed that the simple probe-task was performed faster (*M* = 0.57; *SD* = 0.06) than the complex probe-task (*M* = 0.99; *SD* = 0.26), *t*(29) = -9.25, *p* < 0.001, *r* = -0.736. Moreover, the simple probe-tasks were significantly faster than the complex probe-tasks both in the insight [*t*(24) = -2.53, *p* = 0.018, *r* = -0.247] and non-insight problems [*t*(28) = -2.93, *p* = 0.007, *r* = -0.253].

As we expected, the analysis did not reveal any interaction between the probe type and the stage factor [*F*(1.77,37.21) = 0.5, *P* = 0.59, $\eta_{p}^{2}$ = 0.023], between task type and probe type [*F*(1.7,35.8) = 0.47, *P* = 0.601, $\eta_{p}^{2}$ = 0.022], nor between probe type, task type, and the stage factors [*F*(3.04,63.76) = 0.9, *P* = 0.447, $\eta_{p}^{2}$ = 0.041].

There was a significant main effect of the stage factor [*F*(2,41.95) = 76.04, *P* < 0.001, $\eta_{p}^{2}$ = 0.784] and an interaction between the task type and stage factors [*F*(3.13,65.81) = 31.69, *P* < 0.001, $\eta_{p}^{2}$ = 0.601]. Various task conditions of the probe-task performance revealed different dynamics. The reaction time decreased in the practice trial over time [the first and second stages: *t*(30) = 3.21, *p* = 0.003, *r* = 0.278; the first and third stages: *t*(30) = 4.55, *p* < 0.001, *r* = 0.356], representing a typical learning curve. At the same time, the reaction time increased over time in both insight and non-insight problems [the first and second stages of insight problems: *t*(28) = -3.74, *p* < 0.001, *r* = -0.322; the first and third stages of insight problems: *t*(28) = -6.5, *p* < 0.001, *r* = -0.51; the first and second stages of non-insight problems: *t*(29) = -6.04, *p* < 0.001, *r* = -0.535; the first and third stages of non-insight problems: *t*(29) = -13.22, *p* < 0.001, *r* = -0.764].

*Post hoc* pairwise comparisons with the Holm–Bonferroni adjustment revealed a gradual increase in reaction time values in all conditions. There were significant differences in non-insight problems when performing the simple probe-task between the first and second stages [*t*(29) = -5.46, *p* < 0.001, *r* = -0.454], the first and third stages [*t*(29) = -9.28, *p* < 0.001, *r* = -0.681], and the second and third stages [*t*(29) = -5.26, *p* < 0.001, *r* = -0.416]. The same effect was observed for the complex probe-task in non-insight problems between the first and second stages [*t*(30) = -4.37, *p* < 0.001, *r* = -0.401] and the first and third stages [*t*(30) = -7.2, *p* < 0.001, *r* = -0.587]. Reaction times for both simple and complex probes increased from stage to stage during non-insight problem solving. This may be due to a gradual increase of working memory load by analytical processes and the accumulation of problem-related information over time.

Surprisingly, we observed a stage-to-stage increase of the reaction time for insight problems as well. The reaction time for the simple probe in the first stage of insight problems was smaller than in the second stage [*t*(27) = -4.64, *p* < 0.001, *r* = -0.272] and the third stage [*t*(27) = -4.18, *p* < 0.001, *r* = -0.351]. Similarly, the reaction time for the complex probe in the first stage of insight problems was smaller than in the second stage [*t*(26) = -2.56, *p* = 0.017, *r* = -0.304] and the third stage [*t*(26) = -3.99, *p* < 0.001, *r* = -0.466]. Nevertheless, the reaction times (presumably indicative of working memory load) were generally higher in non-insight problems. However, pairwise comparisons revealed that insight and non-insight problems differ at the second stage [*t*(26) = -2.4, *p* = 0.024, *r* = -0.274] and the third stage [*t*(26) = -5.1, *p* < 0.001, *r* = -0.465] in the simple probe condition and at the second stage [*t*(26) = -2.55, *p* = 0.017, *r* = -0.296] and the third stage [*t*(26) = -3.06, *p* = 0.005, *r* = -0.356] in the complex probe condition. The reaction time for the same probe types in the first stage is equal for the insight and non-insight problems.

The complex probe-task was performed slower both in both insight and non-insight problems but not at the third stage. The reaction times in non-insight problems were different between the probes at the first stage [*t*(28) = -3.68, *p* < 0.001, *r* = -0.344] and second stage [*t*(28) = -2.5, *p* = 0.019, *r* = -0.267]. The same results may be observed in insight problems where the probes were different at the first stage [*t*(24) = -2.82, *p* = 0.009, *r* = -0.277] and second stage [*t*(24) = -2.48, *p* = 0.021, *r* = -0.241]. We argue that simple probes become harder during the later stages of the problem solving process because of the concurrent problem solving processes in the final stage of a solution.

### Discussion

The obtained results generally confirmed our hypotheses. Hypothesis 1, that working memory is necessary for insight problem solving although not to the same degree as for non-insight problem solving, was completely confirmed. We found that working memory load in insight problem solving is higher than the baseline reaction time in practice trials. This leads to a conclusion that while insight problem solving is demanding in terms of working memory, non-insight problem solving is notably more so. While non-insight problem processing includes planning, holding interim calculations in memory, and control; solving insight problems may involve posing and testing hypotheses, problem comprehension, restructuring of a representation, and verification of solutions. These processes are cognitively demanding but are relatively rare, impermanent, and eventual.

Hypothesis 2 was confirmed by the main effect of probe-task type. Probe-task processing occupies a part of working memory capacity during the problem solving process proportionally to task complexity. Comparison of the probe-tasks in the practice trials revealed that these tasks initially differ by their complexity. The complex probe performance during the main problem solving process is slower than the simple probe performance in all problem types. On the one hand, this shows that the probes are performed well and do not crucially distract from the main problem solving process. On the other hand, it can be described as a modality-independent increase in working memory load under the complex condition because we used different modalities in the main problem (the problems were presented textually) and probe-tasks (the probes were presented visually).

Hypothesis 3 was confirmed by the main effect of the stage factor and an interaction of stage and task factors. We found that the patterns of reaction time dynamics are different in various conditions. We observe a clear learning curve in the practice trials for both probes where reaction times decrease from stage to stage. In contrast, working memory load in the insight and non-insight problems prominently increases. The notable difference between the first and third stages in both types of problems demonstrates that cognitively demanding processing accumulates during the problem solving process. Working memory load in the first stage is similar in insight and non-insight problems and is significantly higher than baseline. We theorize that the same processes related to problem comprehension and building a mental model of the problem are implemented at this stage. The further increases to reaction time in non-insight problem solving may be explained by the increasing processing. As mentioned earlier, the same pattern of working memory load is observed in insight problem solving; the closer one gets to insight solution, the more important of a role working memory plays in insight problem solving. Nevertheless, working memory load does not increase to the same degree in non-insight problems.

Unexpectedly, we found that the probe-tasks of different types are performed similarly at the third stage both in the insight and non-insight problems. Based on the qualitative analysis of the experimental sessions, we speculate that participants might have distracted themselves from the probe-tasks to continue engaging in the problem solving process during the later stages of the trial. This distraction might have obscured the difference between the probe-task types. It also means that parallel competition between the two tasks becomes impossible and turns into switching between the tasks. This also indicates the heavy load of working memory during the last stage of the insight solution.

There were some limitations in this experiment. First, increase in reaction time during the last stage could have been confounded by the process of the verbalization required to report the solution. Second, the obtained results do not allow us to draw any definitive conclusions regarding the role of working memory modal-specific systems. Some of such effects were reported to be found in previous studies (Gilhooly and Fioratou, 2009; Chein et al., 2010). We designed and conducted Experiment 2 to overcome the limitations of Experiment 1.

## Experiment 2

To overcome the limitations of the first experiment, we modified the procedure and attempted to isolate the effect of solution verbalization and verification by separating it from the dual task performance. When a participant found a solution for a problem, they were instructed to press a pause button to report the solution and get the experimenter’s response. If the participant’s solution was incorrect, they resumed the dual task performance. Additionally, we attempted to identify the modality of the representational processing in insight problem solving. To do so, we introduced the variable of congruence – whether the problem and the probe-task were of the same modality or not. Representational change in insight problem solving can occur within the modal-specific storage systems while being relatively unaffected by the control system. Visual representational change in insight problems can be processed in the visuo-spatial sketchpad, while verbal restructuring – in the phonological loop. In other words, if the problem and the probe-task are both visual or both verbal – the competition occurs on the storage system level (congruent condition), while if the problem and the probe-task are presented in different format – they do not compete in the same storage systems, only for non-specific control system (non-congruent condition).

The general hypotheses of Experiment 2 were as follows:

(1) Working memory storage systems are involved in both types of problem solving.
(2) There is a modal specificity of working memory storage system load in insight problem solving. Insight problem solving is expected to be more demanding in terms of working memory storage systems, while non-insight problem solving was expected to heavily rely on the control system.
(3) Working memory load varies across different stages of the problem solving process. We expected an increased control system load in non-insight problem solving and an increased storage systems load during the last stages of insight problem solving.

To test these hypotheses, we employed the 2 × 2 × 3 factorial within-subject design. The first factor was primary problem-type with two levels: insight and non-insight. The second factor was a congruence of the primary problem format and the probe-task with two levels: congruent and non-congruent. The stage acted as a grouping variable with three levels: first, middle and last stage of the trial. The response time in the probe-task was measured.

### Method

#### Participants

Participants in the experimental group were 32 volunteers (22 women; age *M* = 21.03; *SD* = 3.01). Participants in the control group were another 32 volunteers (21 women), aged 18–34 (*M* = 21.5; *SD* = 4.86). The majority of the sample consisted of undergraduate and graduate students at Yaroslavl State University. All participants were tested individually; participation was not monetarily compensated.

#### Stimuli

We modified the materials used in the original experiment, introducing two formats of the primary problem – involving visual images and text, as well as two formats of the probe-tasks: visual and text versions as well. These versions were meant to load the corresponding working memory storage system. The congruent condition always featured the problem and the probe-task of the same format (both visual or both text), while the opposite was true for the non-congruent condition.

The two types of the probe-tasks were as follows:

##### The Text Task

Participants were presented with two alternatives: open or closed syllables. They were instructed to respond with the right key every time they saw a closed syllable (e.g., “LON”) and with the left key every time they saw an open syllable (e.g., “PLE”). They were also instructed to perform the task as quickly and accurately as possible.

##### The Visual Task

Participants were presented with two alternatives: obtuse or acute angles. They were instructed to respond with the left key every time they saw an obtuse angle and with the right key every time they saw an acute angle. The instructions were to perform the task as quickly and accurately as possible.

##### Non-insight Text Problems

These problems have clear conditions, solution algorithms and logical answers. Participants know all important operators necessary to find the correct solution and to build the right condition representation. The problem solution is mainly based on the text code. An example of a non-insight text problem: “Three couples went to a party together. One woman was dressed in red, another one – in green and the third one – in blue. The men were also dressed in one of these colors. When all three couples danced, a man in red danced with the woman in blue. “Christina, it is funny, isn’t it? None of us danced with a partner dressed in the same color.” Think about the man dancing with the woman in red. What color is he wearing?”

##### Non-insight Visual Problems

**FIGURE 6:**
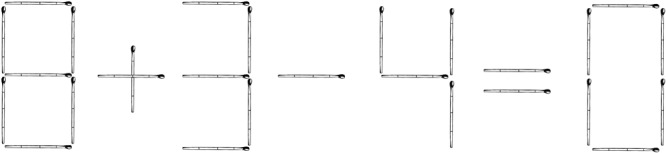
An example of a non-insight visual problem: “Turn inequality into equality by moving one match”.

These problems are similar to non-insight text problems, but the solution is mainly based on the visual code. An example of a non-insight visual problem is the following matchstick problem: “Turn inequality into equality by moving one match: 8 + 3 - 4 = 0” (**Figure 6**).

##### Insight Text Problems

These problems are based on a representational change, but the participant is not aware of the new system of operators. Finding an answer occurs suddenly for solvers and is often accompanied by an emotional response. The solution is mainly based on the text code. An example of an insight text problem: “Sally Lu likes eucalyptus more than pine. She likes electric lighting and does not like to sit by candlelight. Eccentric people evoke more sympathy from her than balanced ones. What do you think is Sally’s profession - an economist or an accountant?”

##### Insight Visual Problems

These problems are similar to insight text problems, but the solution is mainly based on the visual code. An example of an insight visual problem: “Organize 6 identical pencils to get 4 equiangular triangles.”

The problems with an average solution time between 70 to 185 s were selected for the experiment. Participants were not allowed to use notes or write any information down because this would conflict with the probe-task performance. The problems were solved aloud, and participants answered verbally. All the problems are presented in the **Supplementary Materials**.

The control group was included in this study to compare the solution times and solution rates of the problems solved in the dual-task conditions vs. the problems solved without any secondary task. Participants in the control group solved the same set of problems as in the experimental group but without any secondary task (4 insight and 4 non-insight problems).

The experiment was conducted using PsychoPy2 scripts (Version 1.81.02; Peirce, 2008) on the ASUS K55VD computer with a 15.6″ screen.

### Procedure

The procedure used in Experiment 2 was identical to the procedure of the Experiment 1. Each participant solved 8 problems total – one problem trial in each condition presented in random order. The problems were presented at the upper part of the screen; the probe-task stimuli were presented at its center.

The participants were solving problems while performing the probe-tasks continuously the whole time, except for when they were verbally reporting the solution to a problem they were solving. If their proposed solution was incorrect – they resumed performing the secondary probe-task as well as thinking about the problem solution. After the solution to the problem was found, participants had an option to take up to a 1 min break before proceeding to the next problem.

As in Experiment 1, the average response time for the probe-task served as a dependent variable of interest.

### Preliminary Analysis

The data analysis was identical to that from Experiment 1. Thus, each of the 32 participants attempted to solve 8 problems (256 problems in total), but some problem solving trials were excluded: we excluded unsolved problems (took more than 5 min to solve) and problems that were solved in less than 30 s (due to possibility that participant already knew the answer). Besides this, extreme values for the probe-task reaction time above 3 IQR were identified as outliers. Trials with these outliers were excluded from further analysis. Overall, eleven insight problem trials and eighteen non-insight problem trials were excluded from the analysis for those reasons.

Identical to the experimental group, each of the 32 participants in the control group solved 8 problems – one trial in each condition. We used the same criteria for data exclusion. Overall, 51 insight problem trials and 25 non-insight problem trials were excluded from the analysis.

Each problem solving trial was preprocessed and its solution time was split into three equal time intervals as in the Experiment 1. The average reaction time for the probe-task in each of three stages was calculated.

### Results

Obtained results indicated that participants typically solved the majority of the problems (the average solution rate is 70.3%). Similarly, the participants were successfully performing the probe-tasks (87.6% accuracy). This arguably shows that participants were actively engaged in the process and paid sufficient attention and effort to both the primary and secondary tasks.

**FIGURE 7:**
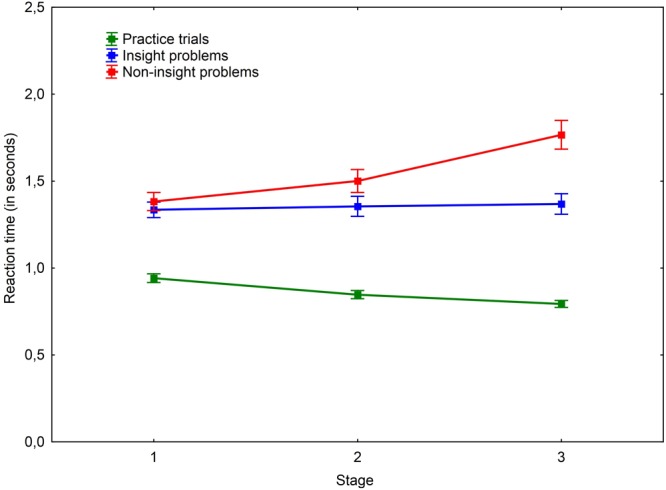
Average dynamics of working memory load in all probe-tasks. Vertical bars denote standard errors.

The average probe-task reaction time in non-insight (*M* = 1.55; *SD* = 0.33) problem solving was greater than in insight problem solving (*M* = 1.35; *SD* = 0.27), *t*(31) = 5.16, *p* < 0.001, *r* = 0.304. Besides, the average probe-task reaction time in insight problems was significantly greater than when the probe-tasks were performed without problem solving (*M* = 0.86; *SD* = 0.11), *t*(31) = 9.08, *p* < 0.001, *r* = 0.748 (**Figure 7**).

We found that solution times in the experimental condition were greater both in insight [*t*(62) = 2.61, *p* = 0.011, *r* = 0.315] and non-insight [*t*(62) = 4.51, *p* < 0.001, *r* = 0.497] problems compared to the control condition. This supports the notion that modally specific probe-tasks affect the problem solving process, however, the probe-tasks were not destructive enough to meaningfully alter the solving process. The solution times of insight problems were significantly greater than that of non-insight problems [*t*(31) = 2.29, *p* = 0.029, *r* = 0.269] in the control group. However, there was no significant difference between insight and non-insight problems solution times in the experimental group [*t*(31) = 1.97, *p* = 0.058, *r* = 0.185]. These results revealed that insight problems were harder than we expected in the control condition, but probe-tasks involvement removed the difference between insight and non-insight problems. The solution rate data showed that insight problems were solved less often. A brief overview of these results can be found in **Table 2**. For a detailed analysis refer to the **Supplementary Table S4**.

**Table 2:** The descriptive statistics of the solution time and the solution rate of the problems in Experiment 2.

	Control group				Experimental group			
	<30 s	>300 s	Solution rate	Solution time, sec (*SD*)	<30 s	>300 s	Solution rate	Solution time, sec (SD)
Insight problems	17 (13.28%)	34 (26.56%)	77 (60.16%)	141.26 (54.08)	4 (3.13%)	32 (25%)	92 (71.88%)	172.4 (40.33)
Non-insight problems	9 (7.03%)	16 (12.5%)	103 (80.47%)	114.44 (41.09)	0	18 (14.06%)	110 (85.94%)	158.01 (36)

*<30 s, a number of problems solved in less than 30 s and excluded from the further analysis. >300 s, a number of problems solved in more than 5 min also excluded from the further analysis.*

#### Problem Type

A repeated measures ANOVA revealed a significant main effect of problem type. The probe-task was performed significantly slower during non-insight problem solving compared to insight problem solving, *F*(1,30) = 37.75, *p* < 0.001, $\eta_{p}^{2}$ = 0.557.

#### Modality Congruence

No significant main effect of modality congruence was revealed. The probe-task average reaction times were equal both in cases when the probe-task was of the same modality as the primary problem and in cases where they were different (e.g., visual problem and a text probe-task), *F*(1,30) = 0.24, *p* = 0.631, $\eta_{p}^{2}$ = 0.008.

#### Problem Stage

A repeated measures ANOVA with Greenhouse–Geisser correction revealed a significant main effect of problem stage, *F*(1.68,50.26) = 19.59, *p* < 0.001, $\eta_{p}^{2}$ = 0.395. A Holm–Bonferroni *post hoc* comparison revealed that the probe-task reaction time was significantly smaller in the first stage (*M* = 1.34, *SD* = 0.04) compared to the middle stage (*M* = 1.42, *SD* = 0.05), while the last stage featured the highest probe-task reaction time (*M* = 1.59, *SD* = 0.07).

#### Problem Type × Modality Congruence Interaction

**FIGURE 8:**
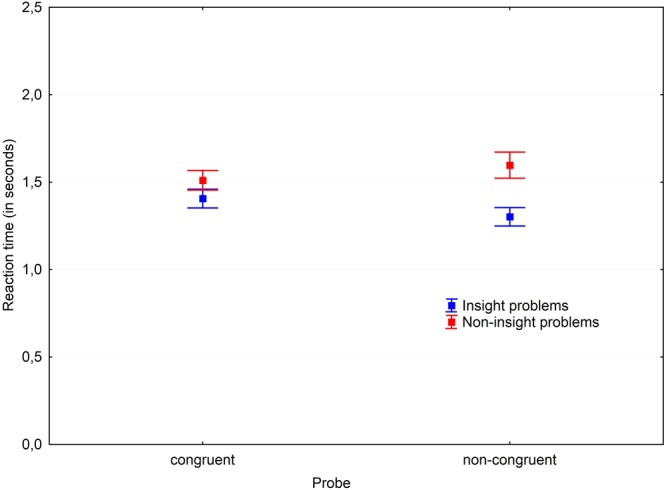
Working memory load in congruent and non-congruent conditions. Vertical bars denote standard errors.

An interaction effect of problem type and modality congruence was found, *F*(1,30) = 8.63, *p* = 0.006, $\eta_{p}^{2}$ = 0.223. A *post hoc* comparison revealed that if the probe-task modality was congruent to the problem modality, its performance became slower for insight problem solving, while it made no difference during non-insight problem solving. It is also notable that probe-task reaction time was significantly slower during non-insight problem solving, compared to insight problem solving only when the probe-task modality was non-congruent to the primary problem (**Figure 8**).

#### Modality Congruence × Problem Stage Interaction

No significant interaction of modality congruence × problem stage was found, *F*(1.88,56.25) = 0.4, *p* = 0.657, $\eta_{p}^{2}$ = 0.01. The probe-task temporal dynamic was approximately the same in both cases, when the problem modality was congruent to the probe-task modality, and when it was not.

#### Problem Stage × Problem Type

A significant interaction effect of problem stage × problem type was found, *F*(2,60) = 33.09, *p* < 0.001, $\eta_{p}^{2}$ = 0.524. A *post hoc* comparison revealed that the probe-task reaction time was initially the same during the first stage for both insight and non-insight problems. However, in the middle stage the probe-task reaction time became significantly slower in non-insight problem solving. The magnitude of change further increased in the last stage. Each consecutive stage in non-insight (but not insight) problem solving featured a significant increase in probe-task reaction time (**Figure 7**).

No significant three-way interaction effect was found, *F*(1.86,55.64) = 1.34, *p* = 0.269, $\eta_{p}^{2}$ = 0.043.

### Discussion

The results of the second experiment indicate that working memory systems are involved in insight and non-insight problem solving processes unequally. Whenever the probe-task and the primary problem were of the same modality, the resource demands were approximately the same (reflected by the same probe-task reaction time) in insight and non-insight problem solving processes. However, in cases when the probe-task and the primary problem were of different modalities – the probe-task during insight problem solving was performed faster than in non-insight problem solving. This leads to a conclusion that non-insight problem solving competes for general resources of working memory – the control system, since competing with the probe-task within the same storage system (phonological loop or visuo-spatial sketchpad) made no difference compared to when the primary problem and the probe-task were processed within separate storage systems. However, it made a substantial difference for insight problem solving – not having both the primary problem and the probe-task processed within the same system at the same time – significantly decreased the average reaction time, and, therefore, reflects better availability of resources in such cases. In other words, the general availability of the control system is more important for non-insight problem solving, while the availability of specific storage systems is more important for insight problem solving. The results suggest that the processing involved in a representation change in insight problem solving occurs on a level as low as the manipulations with the perceptual image of the visual information within the modal-specific storage systems. This falls in line with Duncker’s (1945) ideas regarding insight mechanisms: the solver has to “re-see” the solution (to view the problem from a different angle). Similar findings regarding the importance of modal-specific components can be found in a number of studies which showed that insight problem solving relies on congruency with problem representation storage systems. For example, the nine-dots problem solving performance is positively associated with visual working memory capacity (Chein et al., 2010); heavy visuo-spatial sketchpad load hinders the chess matches problem solving (Robbins et al., 1996); verbal insight problems are solved worse under the phonological loop load (Gilhooly and Murphy, 2005).

Within modality competition and cross-modality competition did not reveal different temporal dynamics over the course of the three stages of problem solving. It seems that although insight and non-insight problem solving processes are different in terms of what working memory components are more crucial for their processing; this difference is equally present during all the stages of the problem solving process. However, the stage-to-stage dynamics without regards to probe-task modality was different for insight and non-insight problem solving processes, replicating the results found in Experiment 1. We observed a gradual increase in the control system load in non-insight problem solving. This might represent the need to keep the results of the intermediate calculations in working memory, as well as the monitoring of the problem solving progress, and the necessity to hold rules and operators in memory. These factors are especially prevalent in non-insight problem solving, but are not as prominently present in insight problem solving because insight solutions mainly require a problem representation shift, which might be less working memory intensive because it does not require the accumulation of explicitly held pieces of information.

The temporal dynamics of working memory load across various stages of insight and non-insight problem solving processes were not affected by whether the probe-task and the primary problem were of the same modality or not. The first reason why this was the case lies in the homogeneity of the initial and final representations of the problem. The problems we used did not require participants to build a problem representation of a different modality in order to achieve the solution. The visual problems required participants to manipulate the visual problem space, while verbal problems revolved around the semantics and the relation between the problem elements. Arguably, if in order to achieve the solution, participants had to switch the modality of the initial problem representation (e.g., verbal to visual), this would have been represented in the results; for example, the visual probe-task reaction time would increase after the initial verbal representation was changed to visual and vice versa. This hypothesis can be tested in future studies. For example, “symmetric problems” (Vladimirov et al., 2016) can be used to investigate this topic, since solving them requires participants to realize that the problem they are facing only appears to be a visual picture reconfiguration, while in reality the problem space represents signs and numbers. The methodological approach we developed (division of the problem into three equal time stages) would likely not be suitable to identify a singular event of the representation change since it is based on averaging a rather large portion of the problem solving session. We plan to supplement this approach by event-related measurements/grouping criteria as well. An impasse and an “aha” moment can serve as markers guiding our data analysis in the future. In particular, Jones (2003) proposed an eye-tracking procedure for identifying the impasse phase. They argue that the moment of the impasse gives way to a more than twofold increase in the fixation duration on certain elements of the problem compared to the average fixation duration prior to that. Identifying the moment of impasse would allow us to test whether the probe-task methodology is consistent with the eye-tracking data.

## General Discussion

In conclusion, we would like to note the technique we used to assess the dynamics of the solution. Despite the popular idea that an insight solution can be divided into various phases, empirical verification of this statement is hard to obtain. Our proposed technique allows one to uncover and probe different phases of the solution separate from each other. This approach lacks disadvantages commonly associated with participant self-reports or an individual differences approach such as: an inability to investigate the micro-dynamics of problem solving; invasiveness – alteration of the natural course of the problem solving process; as well as confound effects of metacognition and memory processes. The main disadvantages are the impossibility of recording the micro-dynamics of problem solving; invasiveness, i.e., influence on the course of the solving process; the low possibility of reflection; the general mechanics of the process; and the influence of metacognitive skills and memory processes in cases of self-reports. The probe-task can act as either a facilitator or a distractor of the problem solving process based on the experimental needs. Besides this, reaction time measurements typically provide a more robust and reliable effect that can benefit the research of working memory during the problem solving process.

It is worth noticing that the probe-task itself in Experiment 1 did not substantially increase the problem (both types) solution times. However, this was the case for Experiment 2 – both insight and non-insight problems were solved slower when performing a dual-task. It is possible that this happened for the very same reason the effects obtained in Experiment 2 were more robust: the combined difficulty level of the problem and the probe-task were likely more appropriate (higher) in Experiment 2.

All in all, both experiments supported the notion that working memory is involved in insight problem solving. Every type of the probe-task used as the secondary task in insight problem solving revealed an increase of reaction time in the dual task condition compared to the single task performance, suggesting a fluctuating impact of the problem solving process on probe-task performance. Working memory in general is involved in both types of problem solving because they share some of the general activities involved in the solving process such as text comprehension, storage of problem elements, holding the interim calculations, attentional control of strategies, and heuristics. Both the control system and storage systems are involved in those general processes. However, the emphasis on either control system or storage systems is different in insight and non-insight problems. While non-insight problem solving is more demanding on the control system, insight problem solving seems to rely on the processing within the modal-specific storage systems to a greater extent. While working memory is typically viewed as a system involved in explicit processing, the fact that working memory (especially the storage systems) plays a role in insight problem solving (that features rather limited conscious self-awareness), supports the idea that working memory is crucial for implicit processing as well (Reber and Kotovsky, 1997; Baars and Franklin, 2003; Soto et al., 2011; Lebed and Korovkin, 2017). Overall, insight problem solving appears to be less demanding on working memory compared to non-insight problem solving, especially if the distinction between control system load and storage systems load is not accounted for.

In terms of the unique contribution of working memory systems, the results indicate that non-insight problems are more demanding on the control system. This could be the case because these problems typically involve more explicit processing, such as progress monitoring, implementation of heuristics, and operations within the problem space. Insight problem solving, on the contrary, involves rejection of the incorrect representations and ineffective rule-sets, which occurs only occasionally and does not require constant monitoring maintained by the control system. This differentiation between the working memory systems involvement was supported by the fact that the probe-task was performed more efficiently if it did not compete for same modality processing as the primary problem – but this was the case only for insight problem solving, not non-insight. Arguably, this notion supports the idea that insight restructuring relies on rather low-level processing that occurs within the working memory storage systems.

All the data regarding the temporal dynamics feature a similar pattern: gradual increase of working memory load in the non-insight problem solving process, but not in the insight problem solving process. This result is in line with our prediction that the solver exerts more and more effort associated with the control system as they progress toward the solution in non-insight problems. The insight problem solving dynamics results were somewhat ambiguous. Results obtained in Experiment 1 revealed a significant increase in working memory load from phase to phase. The results on Experiment 2, however, reveal no such dynamics. Since the procedure in Experiment 2 was modified and participants were not required to perform the probe-task as they were verbally reporting their proposed solution is what might have caused these differences in the results. If this is the case, then the verbalization of the solution in insight problem solving might cooccur with some of the relevant processes contributing to the dynamics in Experiment 1. Such as when the verification of the proposed solution is pronounced verbally.

The lack of observable dynamics in insight problem solving does not speak in favor of the selective forgetting hypothesis (Simon, 1977; Ohlsson, 1992), according to which insight solution involves mere forgetting of the incorrect solutions; if that was the case, one might expect a decrease of working memory load after the incorrect solution was forgotten.

## Conclusion

The proposed probe-tasks technique differs from the traditional distraction paradigm commonly employed in the field. This technique relies on the secondary probe-task reaction time over the course of problem solving, not the problem solution time itself. This paradigm is more suitable for research of working memory load in problem solving.

Insight problem solving is similar to non-insight analytical processing in terms of involvement of working memory resources. However, taking specific functions within working memory into consideration can reveal unique differences between the two problem solving types. Control systems and modal-specific storage systems play a rather different role in insight and non-insight problem solving processes. Insight problems appear to be less demanding on control systems while relying on the availability of modal-specific storage systems in working memory. The working memory demands seem to increase over the problem solving course for non-insight problems, but not for insight problems since they involve less cumulative explicit knowledge acquisition.

Even though identifying the key components involved in insight problem solving can tell us more about the nature of this phenomenon, the control system is crucial for the performance of almost every intellectual activity in humans, therefore, making it rather challenging to isolate its contribution to each problem type individually. Our claim of representational change in insight problem solving occurs within the modal storage systems, should and will be further tested in the future studies.

## Ethics Statement

This study was approved by the Ethics Committee of the Psychology Department of the Yaroslavl State University. All subjects gave written informed consent in accordance with the Declaration of Helsinki.

## Author Contributions

All authors together designed the experiments. AS conducted the first experiment. AC conducted the second experiment. SK and IV wrote the first draft of the manuscript and analyzed the data. All authors were critically involved in the interpretation of the results and in revising the manuscript.

## Conflict of Interest Statement

The authors declare that the research was conducted in the absence of any commercial or financial relationships that could be construed as a potential conflict of interest.

## Supplementary Material

TABLE S1 The list of insight and non-insight problems.

TABLE S2 The results of Experiment 1.

TABLE S3 The results of Experiment 2.

TABLE S4 The solution time and solution rate data for both experiments.
